# Reversal of Increase in Intestinal Permeability by *Mangifera indica* Seed Kernel Extract in High-Fat Diet-Induced Obese Mice

**DOI:** 10.3390/ph13080190

**Published:** 2020-08-11

**Authors:** Pavan Kumar Mujawdiya, Pravesh Sharma, Shashwat Sharad, Suman Kapur

**Affiliations:** 1 Department of Biological Sciences, Birla Institute of Technology and Science, Pilani, Hyderabad Campus, Hyderabad 500078, India; pavanmujawdiya930@gmail.com; 2 Department of Pharmacy, Birla Institute of Technology and Science, Pilani, Hyderabad Campus, Hyderabad 500078, India; praveshvns001@gmail.com; 3 Center for Prostate Disease Research, Department of Surgery, Uniformed Services University of the Health Sciences and the Walter Reed National Military Medical Center, Bethesda, MD 20814, USA; shashwat.sharad.ctr@usuhs.edu

**Keywords:** metabolic syndrome, hepatic fibrosis, tight junction, gut barrier, nesfatin-1

## Abstract

Obesity and hyper-intestinal permeability are interconnected. This study is designed to evaluate the ability of *Mangifera indica* seed kernel extract (MESK) in restoring the intestinal barrier and preventing obesity and associated metabolic complications in a high-fat diet-induced obese mouse model. Four groups of Swiss albino mice: (1) normal diet (ND), (2) high-fat diet (HFD), (3) HFD + Orlistat (100 µg/kg), and (4) HFD + MESK (75 µg/kg), were used to monitor various biochemical parameters associated with metabolic syndrome (glucose, total cholesterol, triglycerides) and body weight in an eight-week-long study. In vivo intestinal permeability was determined by the FITC-dextran method. Interestingly, MESK significantly reduced HFD-induced body weight gain, hepatic lipid accumulation, hepatic fibrosis, hyperglycemia, and dyslipidemia. Additionally, MESK treatment restored the expression of tight junction protein Zonula Occludens-1 (ZO-1) and Claudin-1 and hence prevented increased intestinal permeability induced by a high-fat diet. Moreover, it also increased the expression of potent satiety molecule Nesfatin-1 in the mouse jejunum. Our results, for the first time, establish MESK as a nutraceutical which prevents disruption of the intestinal barrier and thereby intercepts the adverse consequences of compromised intestinal permeability such as obesity, hyperglycemia, dyslipidemia, and systemic inflammation.

## 1. Introduction

**Figure 1:**
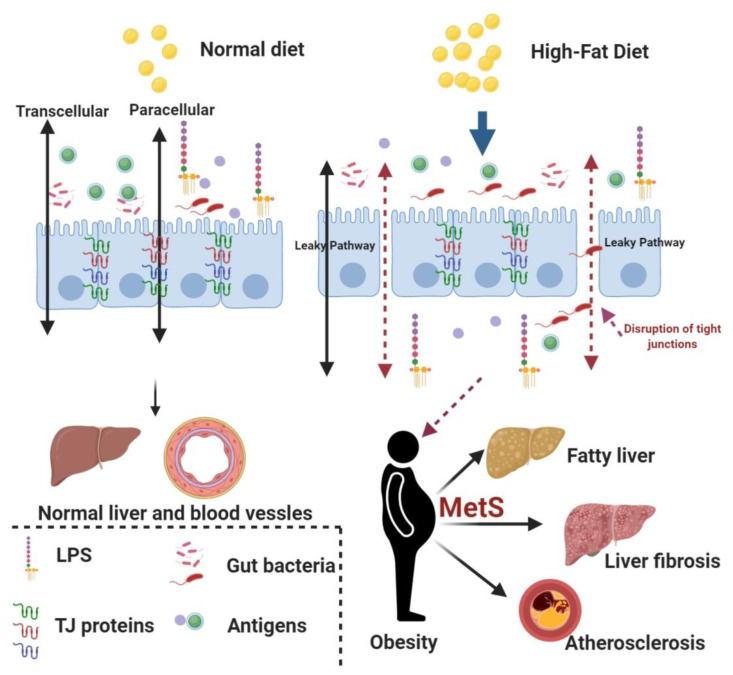
The association between a high-fat diet (HFD), obesity, and obesity-associated complications. High-fat diet promotes the disruption of tight junctions by reducing the expression of key tight junction proteins such as Zonula Occludens-1 (ZO-1), Claudin-1 and Occludin. This compromised intestinal barrier leads to uninterrupted entry of gut bacteria and several bacterial-derived antigens (i.e., lipopolysaccharides (LPS)) to the systemic circulation and promotes a chronic low-grade systemic inflammation in the body. The chronic low-grade inflammation has been considered as a major cause for obesity and other associated complications, such as fatty liver, liver fibrosis, and atherosclerosis (MetS: Metabolic Syndrome).

Intestinal barrier and gut microbiota together modulate the biological and immunological functions of the gut. This plays a crucial role in the development of obesity and associated metabolic complications such as insulin resistance, dyslipidemia, non-alcoholic fatty liver disease (NAFLD), and cardiovascular disorders. Diet and gut microbiota also influence gut permeability [1]. Musso et al. showed that high-fat diet (HFD) increased the serum levels of lipopolysaccharides (LPS), promoted ectopic fat deposition in the liver, and upregulated the levels of several pro-inflammatory cytokines [2]. The higher concentration of LPS in the systemic circulation is attributed to a breach in the intestinal barrier, resulting in reduced expression of key tight junction (TJ)-forming proteins such as Occludin and Zonula Occludens-1 (ZO-1). Downregulation of TJ proteins can severely compromise the integrity of the intestinal barrier and consequently lead to increased intestinal permeability [1,2]. Several other factors such as an imbalance in the gut microbiota composition, consumption of a gluten-rich diet, and higher calorie intake are considered responsible for increased intestinal permeability [3]. It is evident from animal studies that a HFD causes intestinal dysbiosis, chronic low-grade inflammation, and reduces the expression of intestinal peptides and TJ proteins [4]. These physiological changes precede obesity and other related metabolic complications. Further, overexpression of toll-like receptor 4 (TLR-4) initiates a strong inflammatory response, causing disruption in the gut barrier and increased permeability, thus promoting a smooth passage of bacteria-derived components such as LPS, flagellin, and other peptidoglycans from the gut lumen to the systemic circulation [4,5]. The liver is one of the major organs that manifest metabolic complications associated with obesity in the form of NAFLD. NAFLD is the most common cause of chronic liver complications and affects around 20–30% of the global population [6]. This suggests that fat is one of the most important environmental triggers for the development of obesity and NAFLD, although many other factors and genetics also play a major role in the onset and subsequent progression of obesity and related complications [7]. The liver damage due to NAFLD can vary from simple steatosis, where fat droplets deposit into hepatocytes, to non-alcoholic steatohepatitis (NASH), a condition characterized by macrophage infiltration, lobular inflammation, and hepatocyte ballooning [8]. In advanced stages of liver damage, the collagen fibers deposit in the liver and promote the development of liver cirrhosis and hepatocellular carcinoma [8]. NASH is more dangerous than NAFLD because individuals with NASH have higher chances of developing cirrhosis and hepatocellular carcinoma [8]. According to the “multiple-hit” hypothesis, the “first-hit” is provided by abnormal lipid accumulation in the hepatocytes and reduced insulin activity. A continuous sedentary lifestyle could act as a trigger for the “second hit” that causes extensive liver damage characterized by increased lobular inflammation and higher accumulation of extracellular matrix, causing liver fibrosis. The Gut–Liver Axis (GLA) has been shown to play a cardinal role in triggering the “second hit” because a higher intestinal permeability promotes the passage of gut bacteria and bacteria-derived products into the portal circulation, which ultimately leads to a higher inflammatory milieu in the liver. This heightened inflammation in the liver subsequently promotes the transition of simple liver steatosis into advanced stages of liver damage, such as non-alcoholic steatohepatitis (NASH) [9]. Therefore, reducing the HFD-induced increase in intestinal permeability can have beneficial effects in reducing obesity and associated morbidities such as NAFLD, liver fibrosis, and insulin resistance (Figure 1). This also suggests that nutraceutical interventions to prevent disruption of the gut barrier can alleviate/manage the adverse health consequences associated with increased intestinal permeability. Orlistat is a standard anti-obesity drug which reduces intestinal fat absorption due to its inhibitory effect on the intestinal lipase. However, the use of Orlistat is associated with some adverse gastrointestinal health effects such as oily stools, abdominal pain, nausea, and certain liver complications (cholelithiasis and cholostatic hepatitis). In a few patients, Orlistat can also cause liver failure and acute kidney injury [10]. Thus, there is an urgent need to find novel anti-obesity drug molecules with lower side-effects and better therapeutic efficacy than the presently used drugs. Several herbal plants and plant-derived phytochemicals are known to reduce obesity and other related complications. *Mangifera indica*, also called the “king of fruits”, is widely grown in Asia, Africa, and Central America. *Mangifera indica* possesses powerful antioxidant, anti-inflammatory, anti-diabetic, anti-obesity, and anti-cancer properties, and thus can act as a powerful nutraceutical in the treatment of obesity and associated metabolic complications [11,12,13]. The health-promoting effects of *Mangifera indica* are attributed to several bioactive molecules such as Gallic acid, Mangiferin, Quercetin, Chlorogenic acid, and Cinnamic acid [12]. However, no study has as yet explored the correlation between *Mangifera indica* and its protective effects, if any, on the intestinal barrier in a high-fat diet-induced obese mice model. Thus, the aim of the present study is to study whether the extract of *Mangifera indica* seed kernel (MESK) can prevent the gut barrier disruption induced by consumption of HFD and consequently reduce the adverse outcomes of a disrupted gut barrier.

## 2. Materials and Methods

### 2.1. Collection and Preparation of Herbal Extract

50% ethanolic extract of *Mangifera Indica* (MESK) was prepared by mixing 100 g of seed kernel powder into 1500 mL of 50% ethanol in a Pyrex container at 30 °C for 16 h. The extract was filtered using Whatman filter paper (No.1) and stored at 4 °C in an air-tight container. The high-fat diet was prepared by using the procedure described earlier [14].

### 2.2. Phyto-Constituent Analysis of MESK by High Performance Liquid Chromatography (HPLC)

HPLC analysis of MESK was performed on a Shimadzu HPLC system using Kinetex C18 column (150 × 4.6 mm, 100 Å, 5 µm) in an isocratic mode with a flow rate of 0.6 mL/min and detection wavelength of 254 nm. The mobile phase composition for the HPLC run was (A) water + 0.1% Formic acid and (B) methanol (100%). The injection volume for both Gallic acid (100 µg/mL) and MESK (100 µg/mL) was 10 µL [15].

### 2.3. Experimental Animals

**Figure 2:**
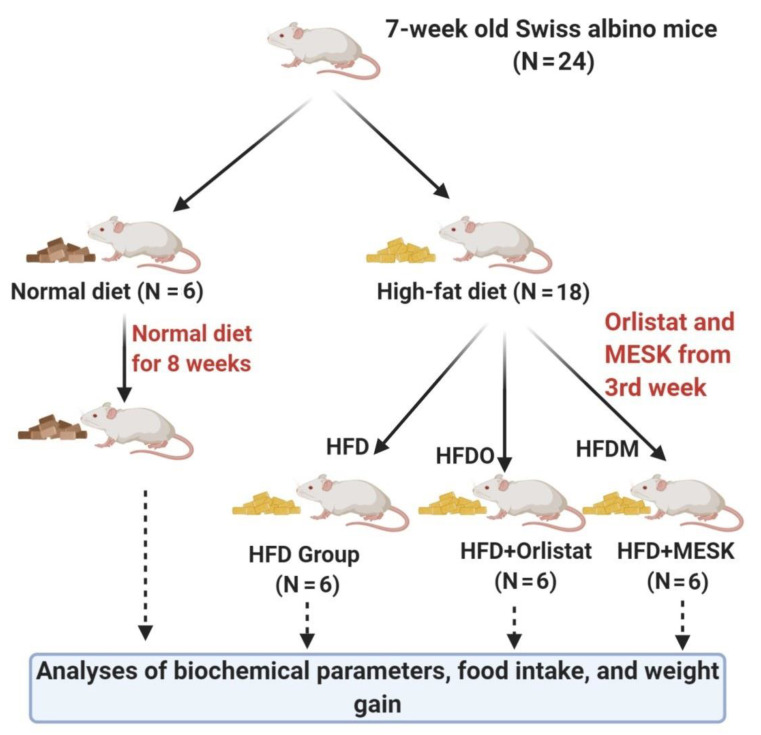
Flow chart representing the study design. The mice were divided into 4 groups after the acclimatization period. The normal diet (ND) mice received a standard chow diet during the complete 8-week study period. Similarly, the HFD group received a high-fat diet during the complete 8-week study period. However, the HFDO (HFD + Orlistat) and HFDM (HFD + *Mangifera indica* seed kernel (MESK)) groups were given respective treatments from the third week onwards until the end of the study.

The animal experiments were performed in accordance with the norms set by the Committee for the Purpose of Control and Supervision of Experiments on Animals (CPCSEA), Government of India, approved Institutional Animal Ethics Committee (IAEC) of Birla Institute of Technology and Science, Pilani, Hyderabad Campus, India. The IAEC approval number for the present study is BITS-HYD/IAEC/2016-10. A group of 24 healthy male Swiss albino mice weighing 20–22 g and aged 7 weeks was obtained from a CPCSEA-approved animal supplier based in Hyderabad. A 12 h light–dark cycle and temperature of 22–23 °C were maintained during the course of the study. Animals were acclimatized for 1 week, during which all groups were provided with ad libitum water and a standard chow diet. After one week, mice were randomly divided into 4 groups as per the protocol outlined in Figure 2.

Group 1 (Normal diet, control group): Animals were kept on a normal standard chow diet for the entire study period of 8 weeks.

Group 2 (HFD alone group): Animals were kept on a HFD for the entire study period of 8 weeks.

Group 3 (HFDO): Animals were kept on a HFD for an initial period of 2 weeks without treatment and then given the standard anti-obesity drug Orlistat for 6 weeks, along with a HFD.

Group 4 (HFDM): Animals were kept on a HFD for 2 weeks without treatment and then given the extract of *Mangifera indica* (MESK) for 6 weeks, along with a HFD.

Diet-induced obesity was generated in mice by feeding them a HFD for 8 weeks (HFD alone group). However, other groups (HFDO and HFDM) received simultaneous doses of the anti-obesity drug Orlistat (100 µg/kg/day) and MESK (75 µg/kg/day) along with a HFD from the third week onwards, until the end of the study. To analyze the biochemical parameters, blood samples from the retro-orbital plexus were drawn into appropriate anticoagulant-containing vials at 0, 2, 4, 6, and 8 weeks. At the end of the study, all animals were euthanized and various tissues such as the liver, intestine, and epididymal adipose tissue were collected and processed for various measurements and stored for further analysis, if required.

### 2.4. Food Consumption, Energy Intake, and Body Weight Measurement

Food intake was measured on a daily basis from the third week onwards by subtracting the amount of food left in the cage grid and spilled food in the cage from the initial weight of the food pellets supplied. The total energy intake was calculated based on the energy density of 3.62 kcal/g for the normal chow diet and 5.18 kcal/g for the HFD diet. Body weight was measured weekly using a digital balance.

### 2.5. Determination of Biochemical Parameters

Biochemical parameters such as glucose, total cholesterol, and triglycerides were analyzed in mouse plasma. Collected blood samples were centrifuged at 3000 rpm for 10 min at 4 °C to obtain clear plasma, which was then transferred in a fresh sterile tube. Plasma levels of glucose, total cholesterol, and triglycerides were measured using kits obtained from Accurex Biomedical Pvt. Ltd., Tarapur, Boisar, Maharashtra, India. Absorbance values were measured using a microplate reader (BioTek Instruments, Winooski, VT, USA).

### 2.6. Epididymal Adipose Tissue (EAT) and Liver Weight Analyses

On completion of the 8-week study period, mice were euthanized and organs (epididymal adipose tissue and liver) were collected and weighed.

### 2.7. Histopathology

For histological examination, the liver and intestine were fixed in 10% buffered Formalin followed by embedding in paraffin. Tissue sections of 5 µm were cut and the sections were stained with Hematoxylin and Eosin to assess hepatocytes’ ballooning and hepatic lipid deposition.

### 2.8. Sirius Red Staining

Collagen deposition in the liver was assessed using the Sirius red staining method described by Segnani et al., with slight modifications [16]. In brief, paraffin-embedded tissue sections of 5 µm were cut, deparaffinized, and rehydrated, as per the standard immunohistochemistry protocols. Tissue sections were then immersed in 0.1% Sirius Red solution (prepared in saturated picric acid) for 1 h to allow saturation of collagen staining, followed by gentle washing in acidified water (0.5% Acetic acid *v*/*v*). The slides were rinsed with normal water and mounted with CC/Mount™ tissue mounting medium (Sigma Aldrich, St. Louis, MO, USA) and the images were captured on a light microscope (Zeiss, Oberkochen, Germany). For quantification of liver collagen, a standard curve of pure collagen was made, and the absolute quantity of collagen was quantified in frozen tissues and expressed as micrograms of collagen/mg of liver tissue [17].

### 2.9. In Vivo Intestinal Permeability Measurement Using the FITC-Dextran Method

The in vivo intestinal permeability was determined by the FITC-dextran method with slight modifications [18]. Mice were fasted for 6 h and an oral dose of 25 mg/kg FITC-dextran was administered. Blood was collected 2 h after the oral dose and FITC-dextran intensity in the serum was analyzed using a fluorescence spectrophotometer (JASCO). A standard curve of known FITC-dextran concentration (diluted in mouse serum) was used to quantify total fluorescence in the serum samples. The excitation and emission wavelengths used were 490 and 520 nm, respectively.

### 2.10. Protein Expression Analysis of ZO-1 and Nesfatin-1/NUCB-2 Using Immunofluorescence

For immunohistochemistry analysis, paraffin-embedded jejunum sections of 5 µm were placed on poly-lysine-coated positive slides. The sections were deparaffinized with 100% xylene (5 min × 3 times) and hydrated through decreasing concentrations of ethanol (95%, 80%, 70%, 50%, and 30%; 2 min each). Finally, the tissue sections were washed with 1× PBS (Phosphate-Buffered Saline), pH 7 (10 min × 2 times) [19]. Antigen retrieval was achieved by immersing tissue sections in Ethylene diamine tetraacetic acid (EDTA) buffer, pH 8 (10 mM EDTA, 0.05% Tween-20) at 95 °C for 20 min. Tissue sections were washed with 1× PBS, pH 7, followed by blocking with 1% Bovine Serum Albumin (BSA) (1 h at room temperature). Tissue sections were incubated with rabbit polyclonal anti-ZO-1 (1:200; sc-10804, Santa Cruz Biotech, Dallas, TX, USA) and mouse monoclonal anti-NUCB-2 (1:200; sc-376947, Santa Cruz Biotech, Dallas, TX, USA) overnight at 4 °C in a humid chamber. The next day, tissue sections were thoroughly washed and incubated with goat anti-rabbit FITC (1:300, Genei, Bengaluru, India) and anti-mouse FITC (1:300; F0257-0.5ML, Sigma) for 2 h at room temperature in a dark chamber. The sections were again thoroughly washed with 1× PBS to remove any non-specific and unbound antibodies and fluorescence was visualized using a confocal laser scanning microscope (Leica DMi8, Wetzlar, Germany).

### 2.11. Immunohistochemical Analysis of Claudin-1

The expression of Claudin-1 in mouse jejunum was analyzed as per the standard immunohistochemistry protocols with slight modifications [20]. In brief, paraffin-embedded jejunum sections (5 µm) were deparaffinized using xylene and rehydrated by a gradient of decreasing concentration of ethanol, as per the protocol mentioned in Section 2.10. After rehydration, antigen retrieval was performed in EDTA buffer, pH 8 (10 mM EDTA, 0.05% Tween-20) at 95 °C for 20 min followed by a washing step in 1× PBS, pH 7. The tissue sections were dipped in 3% solution of hydrogen peroxide in methanol to quench the activity of endogenous peroxidase, and after washing with 1× PBS, sections were blocked using 1% BSA (1 h at room temperature) to reduce non-specific binding. Tissues sections were then incubated overnight with goat polyclonal anti-claudin-1 (1:300; sc-22932, Santa Cruz) at 4 °C in a humid chamber. After the overnight incubation, sections were washed (1× PBS, pH 7) and incubated with anti-goat secondary antibody (1:200) for 2 h at room temperature. The color was developed using a standard diaminobenzidine (DAB): hydrogen peroxide reaction for 3 min in the dark to prevent light-induced oxidation of DAB. The sections were mounted with CC/Mount™ tissue mounting medium (Sigma Aldrich, USA) and the images were captured on a light microscope (Zeiss, Oberkochen, Germany).

### 2.12. Statistical Analysis

All data are expressed as mean ± standard deviation (SD). GraphPad Prism software was used to analyze the data and the t-test was used for statistical analysis. In all comparisons, a *p* < 0.05 was considered to be statistically significant.

## 3. Results

### 3.1. Phytoconstituents in MESK

**Figure 3:**
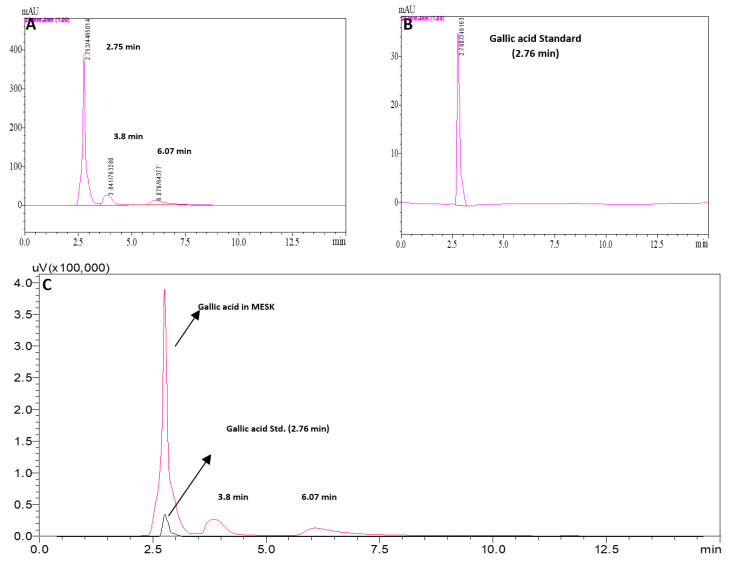
High Performance Liquid Chromatography (HPLC) chromatogram of (**A**) MESK, (**B**) Gallic acid, and (**C**) Overlay of (**A**) and (**B**). HPLC analysis confirmed the presence of Gallic acid as a major bioactive agent in MESK.

The 50% ethanolic extract of *Mangifera indica* (MESK) was characterized using various colorimetric and analytical methods, and the results are published elsewhere [21]. In brief, the total polyphenol, flavonoid, and saponin content of MESK were 187.9 ± 14.5 µg/mg, 160.4 ± 6.2 µg/mg, and 8.3 ± 0.15 µg/mg, respectively. In vitro studies suggested that MESK was a strong inhibitor of α-glucosidase and possesses significant antioxidant properties in 2,2-diphenyl-1-picrylhydrazyl (DPPH) free-radical scavenging assay [21]. We tentatively identified Gallic acid, Ethyl gallate, Cinnamic acid, and Catechin as some of the phytoconstituents in MESK using mass spectroscopy [21]. HPLC analysis confirmed that Gallic acid is one of the major phytoconstituents of MESK (Figure 3).

### 3.2. Effects of Mangifera Indica on Body Weight, Food, and Energy Intake

**Figure 4:**
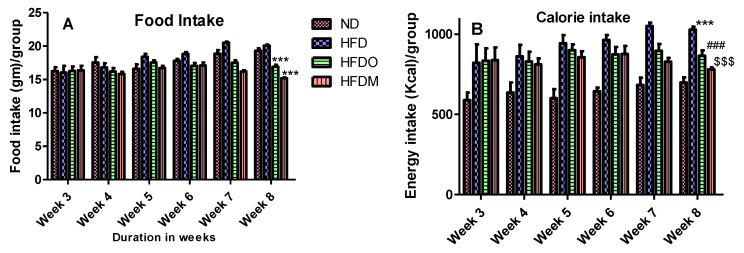
(**A**) Food intake and (**B**) calorie intake during the study (N = 5). The HFDO- and HFDM-treated groups showed significantly lower food and calorie intake at the end of the study. For figure (**A**) *** *p* < 0.001 between HFD and HFDO, and HFD and HFDM. For figure (**B**) *** *p* < 0.01 between ND and HFD, ### *p* < 0.001 between HFD and HFDO, $$$ *p* < 0.001 between HFD and HFDM.

**Figure 5:**
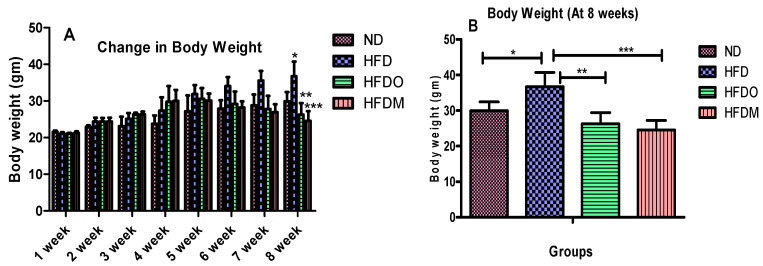
Treatment with MESK reduced the total body weight gain in a high-fat diet-induced obese mouse model. (**A**) Change in body weight during the study and (**B**) comparison of body weight after 8 weeks (N = 5; * *p* = 0.0120 between ND and HFD, ** *p* = 0.0018 between HFD and HFDO, *** *p* = 0.005 between HFD and HFDM). Data are represented as Mean ± standard deviation (SD). (ND: normal diet, HFD: high-fat diet, HFDO: HFD + Orlistat, HFDM: HFD + MESK).

The body weight of all the experimental animals of four groups was the same at the beginning of the study. The food intake was monitored on a daily basis from the third week onwards when the treatment of Orlistat and MESK began. The food intake of the ND and HFD groups were not significantly different but groups treated with Orlistat and MESK showed a significant reduction in food and calorie intake at the end of the study (Figure 4A,B). An 8-week treatment significantly increased the bodyweight of HFD-treated mice (Figure 5A,B). However, a significant reduction in body weight was observed in experimental groups treated with Orlistat and MESK (Figure 5A,B). Our results indicate that MESK is an effective anti-obesity agent and the efficacy in ability to reduce weight was equivalent to that observed with the standard anti-obesity drug Orlistat.

### 3.3. Effect of MESK on the Weight of Epididymal Adipose Tissue (EAT) and Liver

**Figure 6:**
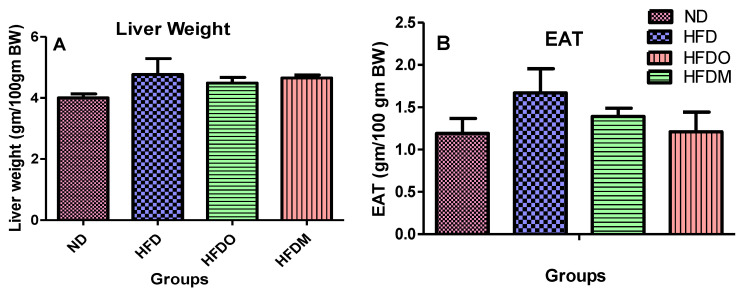
Comparison of liver and epididymal adipose tissue (EAT) weights among various groups. MESK treatment reduced (**A**) the liver weight and (**B**) the epididymal adipose tissue weight. Data are represented as Mean ± SD. (ND: normal diet, HFD: high-fat diet, HFDO: HFD + Orlistat, HFDM: HFD + MESK).

Weights of both EAT and liver were higher in the HFD-treated group (Figure 6A,B) when compared with the normal diet group. However, HFD + Orlistat (HFDO) and HFD + MESK (HFDM) groups showed a reduction in both EAT and liver weights, indicating that MESK treatment is as effective in reducing fat deposition as the standard anti-obesity drug Orlistat.

### 3.4. Biochemical Parameters

**Figure 7:**
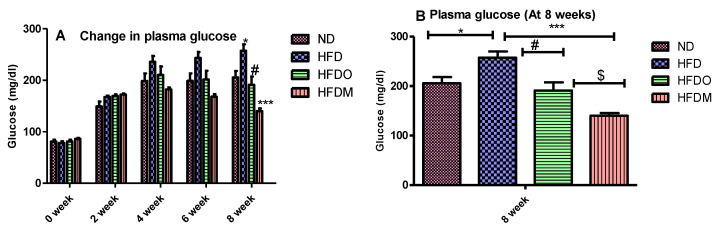
Treatment with MESK reduced the high-fat diet-induced hyperglycemia in the diet-induced obese mouse model. (**A**) Bar graphs representing the changes in glucose levels during the 8-week study period. (**B**) Change in plasma glucose level after the eighth week. (N = 5, * *p* = 0.0114 between ND and HFD, # *p* = 0.0114 between HFD and HFDO, $ *p* = 0.0167 between HFDO and HFDM, *** *p* < 0.001 between HFD and HFDM). Data are represented as Mean ± SD. (ND: normal diet, HFD: high-fat diet, HFDO: HFD + Orlistat, HFDM: HFD + MESK).

**Figure 8:**
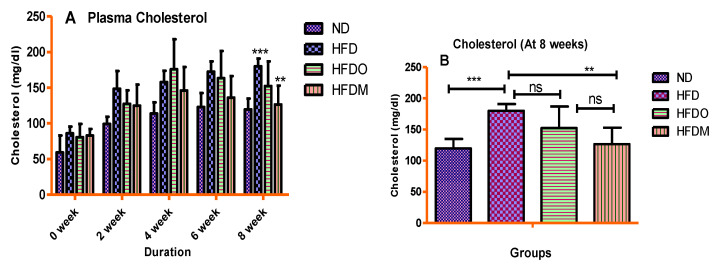
Treatment with MESK ameliorated HFD-induced hypercholesteremia in the diet-induced obese mouse model. (**A**) Change in the plasma cholesterol during the study period and (**B**) change in cholesterol level after the eighth week. (N = 5, *** *p* < 0.001 between ND and HFD, ** *p* = 0030 between HFD and HFDM). (ND: normal diet, HFD: high-fat diet, HFDO: HFD + Orlistat; HFDM: HFD + MESK).

**Figure 9:**
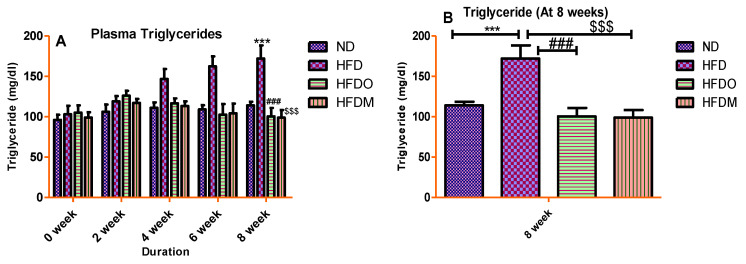
Treatment with MESK reduced the high-fat diet-induced hypertriglyceridemia in the diet-induced obese mouse model. (**A**) Change in the plasma triglycerides during the study period and (**B**) change in triglyceride level after the eighth week. (N = 5, *** *p* < 0.001 between ND and HFD, ### *p* < 0.001 between HFD and HFDO, $$$ *p* < 0.001 between HFD and HFDM). (ND: normal diet, HFD: high-fat diet, HFDO: HFD + Orlistat, HFDM: HFD + MESK).

Plasma values of glucose, total cholesterol, and triglycerides were significantly higher in the HFD-treated group. A gradual rise in the plasma glucose values was observed in the HFD group until the end of the study (Figure 7A). Similarly, HFD treatment significantly increased the plasma values of total cholesterol and triglycerides, indicating that HFD treatment resulted in dyslipidemia in the HFD group (Figure 8A and Figure 9A).

On the other hand, experimental groups given Orlistat and MESK showed a gradual decline in plasma glucose, total cholesterol, and triglycerides once the treatment started from the third week onwards. At the end of the study (8 weeks), MESK significantly reduced all the biochemical parameters (glucose, total cholesterol, and triglycerides) in comparison with the HFD alone group, indicating that MESK is as effective as Orlistat, or more, in lowering HFD-induced hyperglycemia and dyslipidemia (Figure 7B, Figure 8B and Figure 9B).

### 3.5. Effect of MESK on Histological Changes in Liver

**Figure 10:**
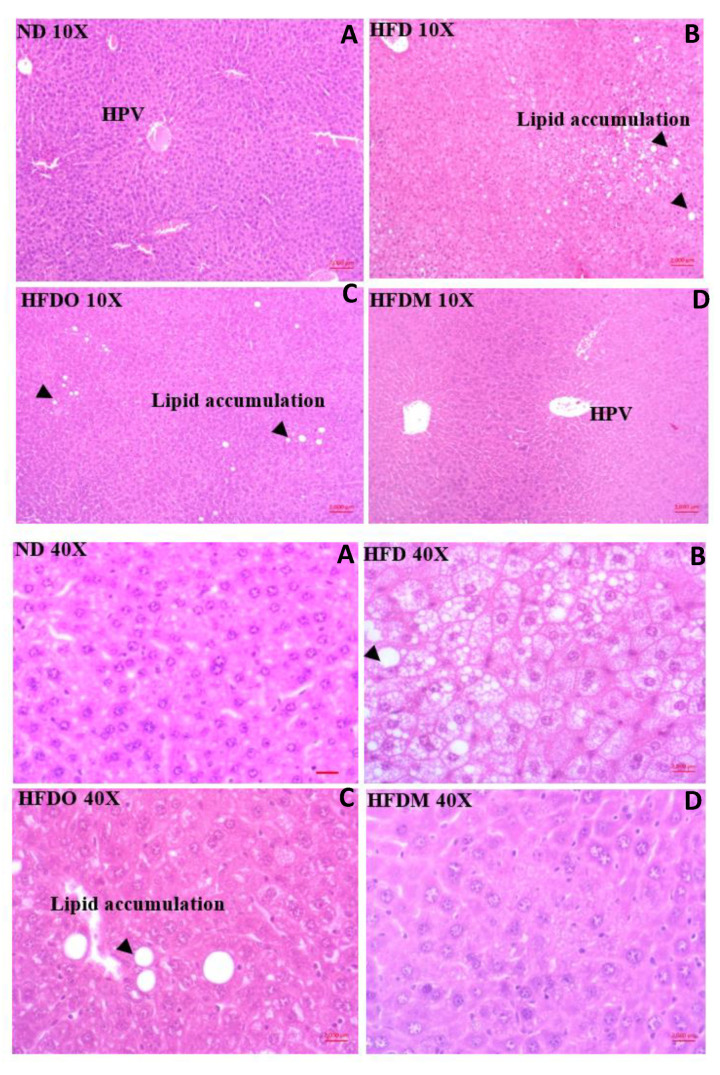
Effect of high-fat diet on fat deposition in the HFD-fed obese mice at 10× and 40× magnifications. Treatment with MESK completely prevented hepatic lipid deposition and hepatic ballooning. (**A**: ND: normal diet, **B**: HFD: high-fat diet, **C**: HFDO: HFD + Orlistat, **D**: HFDM: HFD + MESK). HPV: Hepatic portal vein, Lipid accumulation: fat droplets. Scale bar indicates 2000 µm.

**Figure 11:**
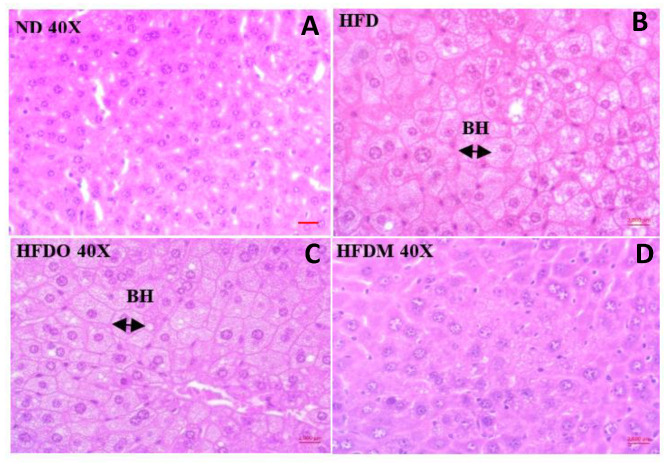
Effect of high-fat diet on hepatic ballooning in the HFD-fed obese mice at 40× magnification. The HFD group was prone to develop hepatic ballooning. Treatment with MESK completely prevented hepatic lipid deposition and hepatic ballooning. (**A**: ND: normal diet, **B**: HFD: high-fat diet, **C**: HFDO: HFD + Orlistat, **D**: HFDM: HFD + MESK). Arrow indicates a higher diameter of ballooned hepatocytes. The ballooned hepatocytes also displayed clear cytoplasm and appeared white in color. Hepatocytes of the normal diet and MESK-treated group showed normal liver architecture and cell size. BH: Ballooned hepatocytes. Scale bar indicates 2000 µm.

The control group receiving a normal diet displayed normal liver histology with no signs of fat deposition and hepatic ballooning (Figure 10A and Figure 11A). The HFD group, however, showed extensive lipid deposition and was prone to hepatic ballooning, indicating liver damage and metabolic stress in the hepatocytes (Figure 10B and Figure 11B). The damaging effects of a HFD on the liver were completely alleviated by treatment with MESK, which displayed normal liver architecture and showed no signs of hepatic ballooning (Figure 10D and Figure 11D). The MESK was even more effective in ameliorating HFD-induced liver damage, as reflected by a higher hepatic ballooning and lipid deposition observed in the Orlistat-treated group (Figure 10C and Figure 11C).

### 3.6. Mangifera indica Extract Prevents Hepatic Fibrosis

**Figure 12:**
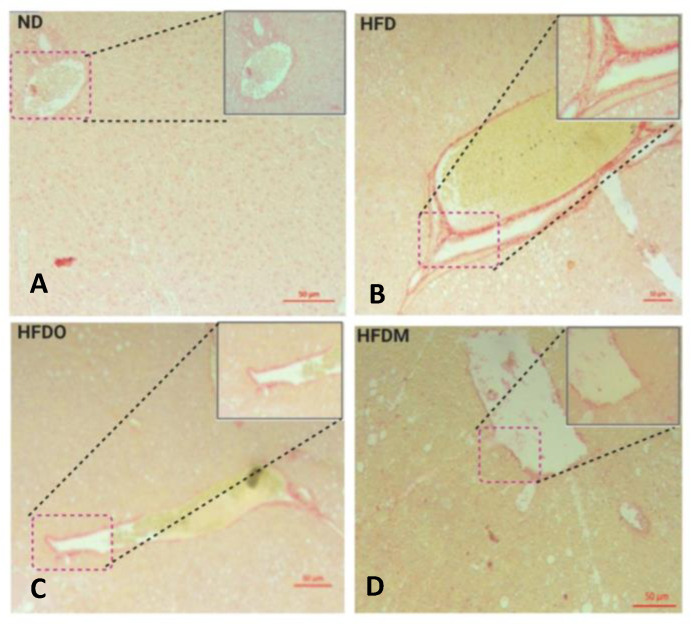
Effect of a high-fat diet (HFD) on hepatic fibrosis. High-fat diet treatment increased collagen deposition in the liver, indicated by a higher intensity of Sirius red staining. This fibrosis was prevented in animals treated with MESK (Magnification 10×). (**A**) Normal diet, (**B**) high-fat diet, (**C**) HFDO: HFD + Orlistat, and (**D**) HFDM: HFD + MESK. Scale bar indicates 50 µm.

**Figure 13:**
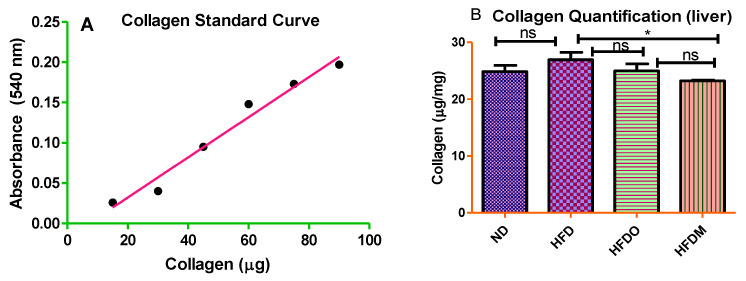
Effect of a high-fat diet on hepatic fibrosis. Treatment with MESK significantly reduced collagen deposition in the liver. (**A**) Collagen standard curve and (**B**) bar graphs representing total collagen as micrograms of collagen/mg of liver tissue (*p* < 0.05; * 0.048 between HFD and HFDM). (ND: normal diet, HFD: high-fat diet, HFDO: HFD + Orlistat, HFDM: HFD + MESK).

The liver of the normal diet-fed group displayed normal collagen deposition, as observed in Sirius red staining (Figure 12A). The collagen fibers appear red in a relatively light yellow or light pink background. Mice fed with a HFD developed liver fibrosis, as reflected by increased hepatic collagen deposition measured by Sirius red staining in the liver (Figure 12B). Treatment with MESK significantly reduced collagen deposition in the liver (Figure 12D) and prevented liver fibrosis. A collagen standard curve ranging from 10 to 50 µg/mL was prepared (Figure 13A) and collagen in the mouse liver was quantified and expressed as micrograms (mg) of collagen/mg of liver tissue (Figure 13B). Our results indicate that treatment with MESK significantly reduced collagen deposition in the liver and prevented liver fibrosis.

### 3.7. Assessment of Intestinal Permeability

**Figure 14:**
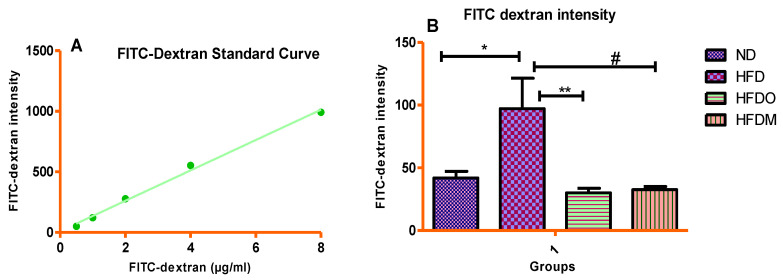
(**A**) FITC-dextran standard curve and (**B**) comparison of intestinal permeability as measured by the FITC-dextran method. Treatment with MESK and Orlistat reduced the intestinal permeability to near normal levels. (* *p* = 0.0183 between ND and HFD, # *p* = 0.0101 between HFD and HFDM, ** *p* = 0.0091 between HFD and HFDO). (ND: normal diet, HFD: high-fat diet, HFDO: HFD + Orlistat, HFDM: HFD + MESK).

In vivo intestinal permeability was measured using the FITC-dextran method, as described in Section 2.9. It was observed that HFD treatment significantly increased the appearance of FITC-dextran in serum, indicating a compromised/breached gut barrier (Figure 14B). This HFD-induced increase in intestinal permeability was ameliorated by treatment with Orlistat and MESK, indicating that experimental groups receiving the intervention were protected from HFD-induced gut-barrier disruption (Figure 14B).

### 3.8. Expression of Zonula Occludens-1 in the Small Intestine

**Figure 15:**
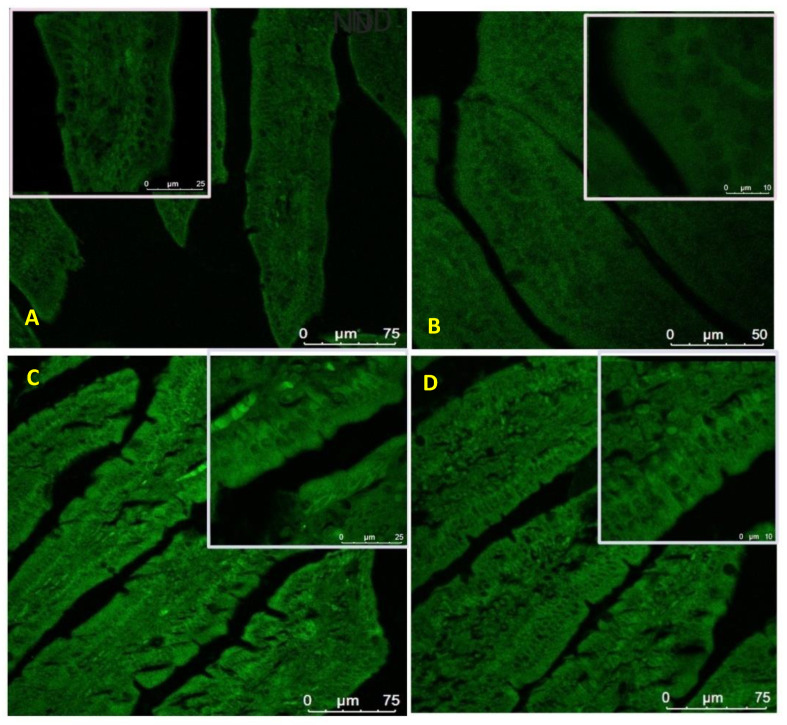
The impact of high-fat diet treatment on ZO-1 expression in mouse jejunum. The HFD-fed group showed lower expression of ZO-1, a key tight junction protein. Treatment with MESK and Orlistat restored the expression of ZO-1 (**A**: ND: normal diet, **B**: HFD: high-fat diet, **C**: HFDO: HFD + Orlistat, **D**: HFDM: HFD + MESK).

Treatment with the HFD diet was found to disrupt the integrity of tight junctions by reducing the expression of key tight junction proteins. Due to the critical role played by ZO-1 in tight junction formation and normal intestinal barrier functioning, we focused our analysis on the expression of ZO-1. The expression of ZO-1 was studied using immunofluorescence and our results show that HFD treatment reduced the expression of ZO-1 in mouse jejunum (Figure 15B). However, treatment with both Orlistat and MESK reversed the adverse effects of HFD by restoring the expression of ZO-1 (Figure 15C,D).

### 3.9. Expression of Claudin-1 in the Small Intestine

**Figure 16:**
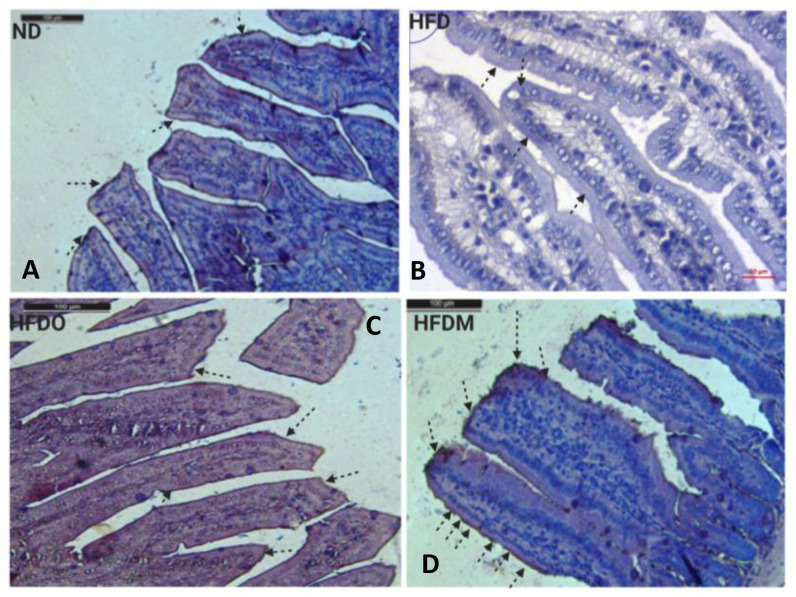
The effects of MESK supplementation on Claudin-1 expression in mouse jejunum (Magnification 20×). (**A**) Normal diet, (**B**) high-fat diet, (**C**) HFDO: HFD + Orlistat, and (**D**) HFDM: HFD + MESK. HFD-fed mice displayed reduced expression of Claudin-1 in both villus tip and villus border (arrows). Treatment with MESK and Orlistat restored the expression of Claudin-1 on both the villus tip and villus border (arrows). Scale bar indicates 10 µm.

Claudin-1 expression in the intestine was measured using standard DAB-H_2_O_2_ immunohistochemistry protocols. Claudin-1 is tetraspan protein present on the tight junctions and helps in pore formation between the adjacent epithelial cells and controls paracellular permeability. Owing to its critical role in paracellular permeability, we analyzed the expression of Claudin-1 in mouse jejunum. It was observed that HFD reduced the expression of Claudin-1 in jejunum (Figure 16B). However, treatment with both Orlistat and MESK restored the expression of Claudin-1 in the jejunum (Figure 16C,D). Taken together, MESK and Orlistat restored the normal intestinal expression of both ZO-1 and Claudin-1 in the jejunum, and hence prevented the damaging effects of HFD on the intestine and restored normal intestinal permeability. 

### 3.10. Expression of Nesfatin-1/NUCB-2 in the Small Intestine

**Figure 17:**
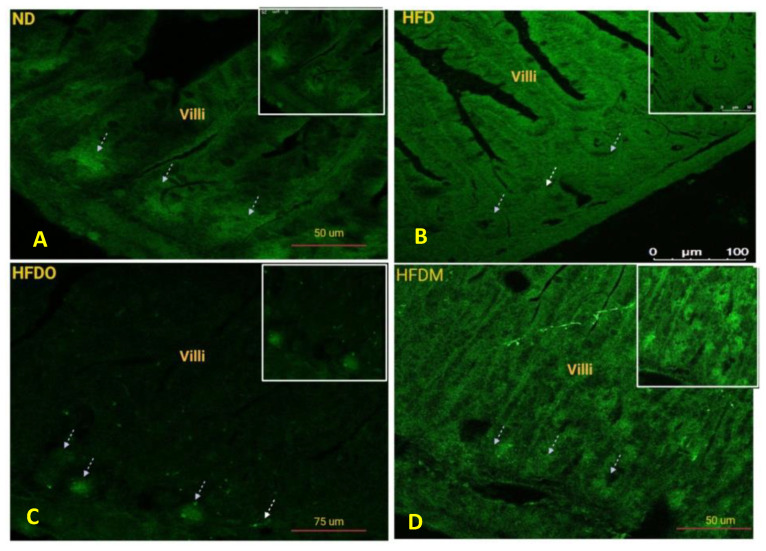
The impact of high-fat diet treatment on Nesfatin-1/NUCB-2 expression in mouse jejunum using immunofluorescence. HFD-fed mice showed lower expression of satiety molecule Nesfatin-1 in the mouse jejunum (arrows). Treatment with MESK and Orlistat increased the expression of Nesfatin-1 (arrows). (**A**) ND: normal diet, (**B**) HFD: high-fat diet, (**C**) HFDO: HFD + Orlistat, and (**D**) HFDM: HFD + MESK.

The expression of Nesfatin-1 in the mouse jejunum is shown in Figure 17. It was observed that treatment with HFD reduced the expression of Nesfatin-1 in the crypts of the jejunum (Figure 17B). However, Orlistat and MESK treatment increased the expression of Nesfatin-1 in the mouse jejunum (Figure 17C,D). Since Nesfatin-1 is a satiety hormone, a higher expression of Nesfatin-1 in the jejunum may be involved in a decrease in weight gained in both HFDO and HFDM groups.

## 4. Discussion

**Figure 18:**
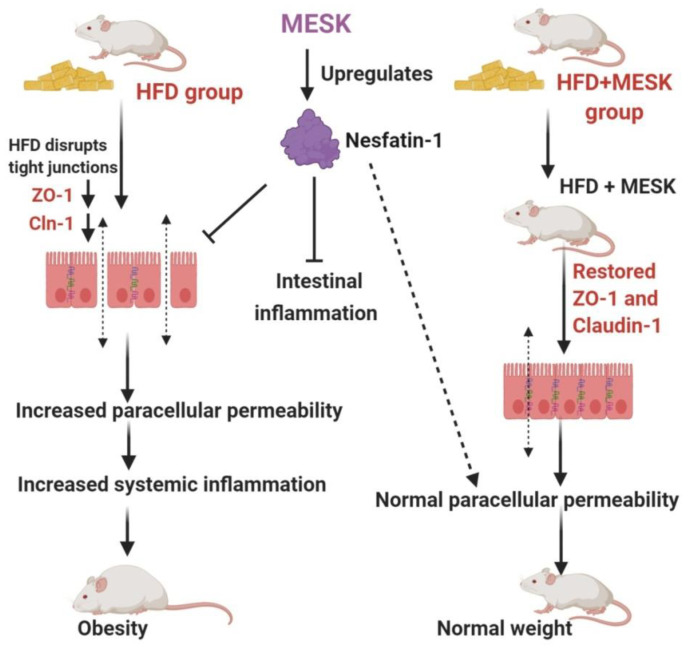
Flow chart explaining the anti-obesity and gut barrier-protective effects of MESK. MESK upregulated the expression of Nesfatin-1, which in turn reduced food intake and also the disruption of the gut barrier and prevented obesity and associated metabolic complications.

Recent decades have seen an unprecedented rise in lifestyle-related disorders such as obesity, cardiovascular disorders, non-alcoholic fatty liver syndrome, and insulin resistance, collectively referred to as the metabolic syndrome. Reduced physical activity due to mechanized transport, urbanization, and a shift towards energy-dense foods have been recognized as the chief contributing factors for this global health epidemic [8,22]. However, at the molecular level, the main triggering factor for metabolic syndrome is a persistent, systemic, chronic low-grade inflammation. It has been demonstrated that inflammation in the gut precedes obesity, insulin resistance, and fat accumulation [23]. The intestinal barrier in the gut separates the gut lumen and the systemic circulation. Moreover, the intestinal barrier also harbors several functional components that comprise muscular, neurological, immunological, and humoral elements. Intestinal permeability is a functional property of the intestinal barrier that regulates the passage of molecules from the gut lumen to the systemic circulation [18]. Normal intestinal permeability observed in healthy subjects is devoid of inflammation and impaired intestinal functions. However, impaired intestinal permeability disturbs the intestinal homeostasis and allows uninterrupted passage of various antigens, including LPS derived from the gut microbiota, to the systemic circulation, leading to a systemic immune response and chronic low-grade inflammation. This chronic low-grade inflammation caused by increased LPS leakage to the systemic circulation is sufficient to induce obesity in animal models. It has been shown that mere infusion of LPS in mice can trigger obesity and insulin resistance. Due to this reason, a higher intestinal permeability is considered as a causative factor for obesity and associated metabolic syndrome [24,25,26]. Over the last decade, several studies in animal models and human subjects have observed a positive correlation between obesity and intestinal permeability [27]. Increased intestinal permeability also adversely affects the normal functioning of the liver. For instance, increased intestinal permeability observed in genetically obese *ob*/*ob* and *db*/*db* mice contributes to portal endotoxemia, thus making the hepatic stellate cells (HSCs) of genetically obese *ob*/*ob* and *db*/*db* mice more sensitive for various fibrogenic and inflammatory responses than HSCs derived from the lean animals. This suggests that intestinal hyperpermeability is a causative factor of liver fibrosis and hepatic inflammation [28]. An increased intake of a high-fat diet can significantly reduce the expression of tight junction proteins and hence, increases the intestinal permeability by damaging the intestinal barrier. Studies have shown that mice fed with a HFD displayed increased intestinal damage compared to mice fed a low-fat diet [29]. These observations suggest a close association between intestinal permeability and onset of obesity and other metabolic complications such as hyperglycemia, insulin resistance, and dyslipidemia. Therefore, reducing the intestinal damage caused by a HFD by using nutraceutical and herbal interventions can be a promising intervention strategy to manage the adverse health consequences of increased intestinal permeability. In the present study, we have shown that 50% ethanolic extract of *Mangifera indica* seed kernel (MESK) prevented weight gain, hyperglycemia, dyslipidemia, hepatic ballooning, hepatic lipid accumulation, hepatic fibrosis, and an increase in intestinal permeability induced by HFD in Swiss-albino mice. As shown in Figure 10, HFD treatment increased the hepatic lipid accumulation, while the MESK-treated group displayed a reversal of lipid accumulation and the ameliorating effects were superior in comparison with the standard anti-obesity drug Orlistat (Figure 10). Treatment with MESK and Orlistat inhibited the lipid accumulation, as reflected by lower numbers of lipid globules in the liver (Figure 10B–D). We have also shown that animals treated with MESK displayed normal liver histology, and hepatocytes showed no signs of hepatic ballooning (Figure 11D) as opposed to that seen in animals fed with HFD (Figure 11B). The HFD-fed group also displayed increased liver collagen deposition as reflected by the increased intensity of Sirius red staining observed in the HFD-fed group (Figure 12B). Changing dietary habits and reduced physical activity has increased the incidences of NAFLD to an alarming proportion, and presently, 25% of the world’s population is affected by NAFLD. It is pertinent to note that the onset of NAFLD is considered as the hepatic response of metabolic syndrome and in the absence of any preventive measures, NAFLD can progress towards other irreversible liver complications such as NASH, liver fibrosis, cirrhosis, and hepatocellular carcinoma [30,31]. Liver is one of the principal organs involved in lipid metabolism, and abnormal functioning of cellular pathways involved in lipid deposition and lipid metabolism facilitates the accumulation of lipid in hepatocytes, leading to excessive lipid deposition in the liver [32]. It has been observed that rearrangement of various cytoskeleton proteins, deposition of fat droplets in hepatocytes, and deformity in the structure of the endoplasmic reticulum are some of the major causes behind hepatic ballooning [33]. Normal intestinal permeability is maintained by tight junctions, which are multi-protein complexes comprising of several proteins, namely Zonula-Occludens, Claudin, and Occludin [34]. As discussed above, increased intestinal permeability caused by HFD can trigger obesity, NAFLD, and insulin resistance [1,2,3,4,5,6,7,8,9,10,11]. Here, we have demonstrated that both MESK and Orlistat prevented the disruption of tight junctions caused by HFD. The HFD-treated group showed lower expression of both ZO-1 and Claudin, two crucial tight junction proteins needed for normal assembly of the intestinal tight junctions. We showed that treatment with both Orlistat and MESK increased the expression of ZO-1 in the mouse jejunum (Figure 15C,D). Moreover, Orlistat and MESK treatment also prevented HFD-induced reduction in the expression of another key tight junction protein, Claudin-1 (Figure 16C,D). This indicates that both Orlistat and MESK prevent disruption of tight junctions induced by HFD via the upregulation of ZO-1 and Claudin-1, critical tight junction proteins involved in maintaining a healthy intestinal barrier and normal intestinal permeability. This reversal of increased intestinal permeability in the HFDO- and HFDM-treated groups is reflected by the lower FITC-dextran intensity observed in the serum of the HFDO and HFDM groups (Figure 14B). Recently, Zhang et al. reported that Orlistat prevented gut barrier disruption in HFD-fed *Nile tilapia* by upregulating the expression of Claudin-3, a tight junction protein [35]. We also measured the expression levels of Nesfatin-1, a potent anti-obesity and anti-inflammatory peptide, in the jejunum. Nesfatin-1, an 82 amino acid peptide hormone derived from post-translational processing of the N-terminal of NUCB-2, is a potent anorexigenic hormone and reduces appetite and weight gain in both mice and humans [36,37]. Nesfatin-1 is expressed in several peripheral organs such as the liver, stomach, kidney, lungs, small intestine, large intestine, and the heart [36,37,38]. It has been demonstrated that HFD reduced serum levels of Nesfatin-1 in male C57BL/6J mice [39]. The expression of Nesfatin-1 in the small and large intestine plays an important role in nutrient absorption and gastrointestinal functions [40]. Recent studies have shown that Nesfatin-1 prevented intestinal damage by upregulating ZO-1 and Claudin-3. It also inhibited the activity of the NF-κB-65 pathway and helped in maintaining the oxidant/antioxidant system in the intestine [41]. We postulate that increased expression of Nesfatin-1 is responsible for reduced weight gain in HFDO and HFDM groups and the anti-obesity and intestinal protective effects of MESK are due to increased expression of Nesfatin-1 caused by MESK (Figure 18). The present study demonstrated that MESK has the ability to reverse HFD-induced increases in intestinal permeability by upregulating the expression of key TJ proteins ZO-1 and Claudin-1 (Figure 15D and Figure 16D). We have also demonstrated that MESK and Orlistat increased the expression of Nesfatin-1 in the mouse jejunum (Figure 17C,D). Since Nesfatin-1 is a powerful satiety signal, a higher expression of this peptide hormone could be the reason behind the reduced weight gain (Figure 5A,B) observed in HFDO and HFDM groups, though the involvement of other satiety hormones cannot be ruled out. Orlistat acts by reducing the activity of intestinal lipase, and a recent study by Olszanecka-Glinianowicz has shown that long-term treatment (8 weeks) with Orlistat significantly increased the plasma levels of anorexigenic hormones glucagon-like peptide-1 (GLP-1) and Peptide YY (PYY) and the rise in the levels of these satiety hormone levels was twice those of the placebo group [42]. In our study, we observed a rise in the expression of satiety hormone Nesfatin-1 in the jejunum of Orlistat- and (Figure 17C) MESK-treated groups (Figure 17D). Tian et al. demonstrated that increased expression of Nesfatin-1 in the gastro-intestinal tract was associated with body mass and energy metabolism [43]. Nesfatin-1 also displayed anti-inflammatory activity due to its inhibitory effects on the cyclooxygenase-2 (COX-2) pathway [44]. Intestinal inflammation has been shown to increase the intestinal permeability by remodeling the tight junctions [45]. For instance, pro-inflammatory cytokines such as TNF-α can cause time-and-dose-dependent increases in the tight junction permeability and reducing the TNF-α by dietary interventions prevented a TNF-α-induced rise in intestinal permeability [46]. Based on the experimental findings and available scientific evidences, we believe that MESK treatment upregulated the expression of Nesfatin-1, which in turn reduced the intestinal inflammation, disruption of tight junctions, prevented systemic inflammation, and hence, reduced the weight gain in the MESK-treated group (Figure 18). The present study demonstrates that *Mangifera indica* extract (MESK) reversed the HFD-induced intestinal hyper-permeability and also reduced the hyperglycemia and dyslipidemia induced by HFD. As discussed above, the reversal of “leaky-gut” will also prevent the onset of chronic low-grade inflammation by preventing the undesirable passage of bacteria-derived antigenic components into the systemic circulation. Combined together, these data indicate that MESK extract can be a potent nutraceutical that can be used for the prevention/treatment of obesity and obesity-associated metabolic complications.

## Abbreviations

BSA	Bovine Serum Albumin
EAT	Epididymal Adipose Tissue
EDTA	Ethylenediamine Tetra Acetic acid
FITC-dextran	Fluorescein Iso-Thio-Cyanate Dextran
GLP-1	Glucagon-Like Peptide-1
HB	Hepatocyte Ballooning
HFD	High Fat Diet
HPV	Hepatic Portal Vein
LPS	Lipopolysaccharides
MESK	50% Ethanolic Extract of *Mangifera indica* seed kernel
NAFLD	Non-Alcoholic Fatty Liver Disease
NUCB-2	Nucleobindin-2
PYY	Peptide YY
TLR	Toll-Like Receptor
ZO-1	Zonula occludens-1
